# UCSC Cell Browser: visualize your single-cell data

**DOI:** 10.1093/bioinformatics/btab503

**Published:** 2021-07-09

**Authors:** Matthew L Speir, Aparna Bhaduri, Nikolay S Markov, Pablo Moreno, Tomasz J Nowakowski, Irene Papatheodorou, Alex A Pollen, Brian J Raney, Lucas Seninge, W James Kent, Maximilian Haeussler

**Affiliations:** Genomics Institute, University of California Santa Cruz, Santa Cruz, CA 95064, USA; Department of Biological Chemistry, University of California, Los Angeles, CA 90095, USA; Division of Pulmonary and Critical Care, Feinberg School of Medicine, Northwestern University, Chicago, IL 60611, USA; EMBL-EBI European Bioinformatics Institute, Wellcome Genome Campus, Hinxton, Cambridge CB10 1SD, UK; The Eli and Edythe Broad Center of Regeneration Medicine and Stem Cell Research, University of California San Francisco, San Francisco, CA 94143, USA; Department of Anatomy, University of California San Francisco, San Francisco, CA 94143, USA; Department of Psychiatry and Behavioral Sciences, University of California San Francisco, San Francisco, CA 94143, USA; Chan Zuckerberg Biohub, San Francisco, CA 94158, USA; EMBL-EBI European Bioinformatics Institute, Wellcome Genome Campus, Hinxton, Cambridge CB10 1SD, UK; The Eli and Edythe Broad Center of Regeneration Medicine and Stem Cell Research, University of California San Francisco, San Francisco, CA 94143, USA; Department of Neurology, University of California San Francisco, San Francisco, CA 94143, USA; Genomics Institute, University of California Santa Cruz, Santa Cruz, CA 95064, USA; Genomics Institute, University of California Santa Cruz, Santa Cruz, CA 95064, USA; Department of Biomolecular Engineering, University of California Santa Cruz, Santa Cruz, CA 95064, USA; Genomics Institute, University of California Santa Cruz, Santa Cruz, CA 95064, USA; Genomics Institute, University of California Santa Cruz, Santa Cruz, CA 95064, USA

## Abstract

**Summary:**

As the use of single-cell technologies has grown, so has the need for tools to explore these large, complicated datasets. The UCSC Cell Browser is a tool that allows scientists to visualize gene expression and metadata annotation distribution throughout a single-cell dataset or multiple datasets.

**Availability and implementation:**

We provide the UCSC Cell Browser as a free website where scientists can explore a growing collection of single-cell datasets and a freely available python package for scientists to create stable, self-contained visualizations for their own single-cell datasets. Learn more at https://cells.ucsc.edu.

**Supplementary information:**

Supplementary data are available at *Bioinformatics* online.

## 1 Background

Single-cell RNA-seq assays allow for the exploration of gene expression at unprecedented detail, for surveying cellular diversity in organs (Soraggi *et al.*, 2021) or characterizing cellular states in development (de Soysa *et al.*, 2019) and disease (Kathiriya *et al.*, 2021). As a result, the number of publications using a single-cell RNA-seq assay has grown exponentially since 2010 (Supplementary Fig. S1). This growth has created a need for interactive tools that allow scientists to explore these complex datasets at a high-level before diving deeper into computational analysis with collaborators and for sharing them with the scientific community after publication.

Analysis of single-cell datasets typically begins with metadata and an expression matrix, which are then taken through a few standard steps: (i) normalization, (ii) dimensionality reduction, (iii) clustering, (iv) marker gene identification and cluster labeling and (v) visualization and sharing. Expression matrices are often normalized, trimmed or batch-corrected. They are mapped from many dimensions to just two as *x*, *y* coordinates by dimensionality reduction algorithms like tSNE (van der Maaten and Hinton, 2008) and UMAP (Dorrity *et al.*, 2020). These algorithms attempt to retain much of the original cell similarity structure. The expression matrix is also fed to a clustering algorithm in an effort to find groups of similar cells. Clusters are usually manually annotated using known cell type markers. In most research groups, cluster annotation happens by visualizing dimensionality reduction plots of well-known marker genes in the UCSC Cell Browser, Seurat, Scanpy or one of many other tools (see below).

## 2 Features

**Fig. 1.**
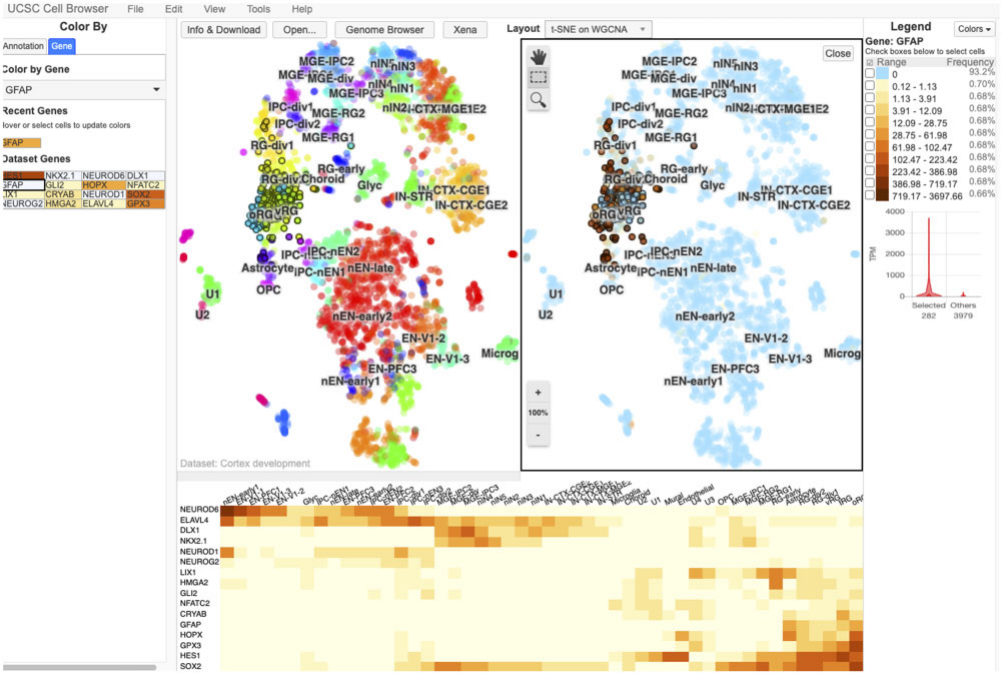
The UCSC Cell Browser interface showing a dataset focused on human cortex development. In the center of the screen, the primary layout view has been split into two vertical panes. The left pane shows the cells in this dataset colored by their metadata value for the field “WGCNACluster” (e.g. RG-div1). The right side shows the same cells colored by their expression of the gene HES1, those with a higher expression being colored dark red. On the far right side of the screen, there is a legend outlining how the colors are associated with expression bins; below this is a violin plot of this gene expression in a set of selected cells (those outlined in black in the center of the screen) versus all other cells in the dataset. On the far left side of the screen, you can see a list of metadata annotation fields available for this dataset. Each one can be clicked to color the plot by the values in that field. Below the two vertical panes, a heatmap shows 16 different genes that the authors of this dataset consider important “dataset genes”

The UCSC Cell Browser allows scientists to visualize the output of these single-cell analysis methods. Its primary display is a two-dimensional scatter plot, most commonly the output of tSNE or UMAP dimensionality reductions (Fig. 1, upper panel center). As a single dataset may produce several *x*, *y* coordinates, scientists can switch between different layouts. Scientists can pan and zoom along the plane, similar to navigating within geographic map viewers. Cells can be colored based on provided annotations (e.g. cell type, age) or by gene expression using several built-in color palettes. The display can be split into two panes offering a side-by-side view allowing comparison between metadata attributes or genes (View > Split; Fig. 1, upper panel). Cells can be selected, either by visually selecting groups or by combining one or more metadata-based filters (Edit > Find Cells). Cell identifiers can be exported for use in other analyses (Edit > Export Cells). 

In addition to a two-dimensional scatter plot, the tool provides other ways to explore single-cell data. Datasets are typically accompanied by a curated list of ‘dataset genes’ for coloring the scatter plot. When enabled, the heatmap view shows the expression of these dataset genes across labeled clusters (View > Heatmap; Fig. 1, lower panel). After selecting cells, histograms showing the distribution of metadata values can be shown as well as violin plots comparing the expression of a gene in the selection against all other cells in the dataset or user-defined ‘background’ cells.

The UCSC Cell Browser can be used to display any high-dimensional data, such as single-cell ATAC-seq data (Supplementary Fig. S2) or bulk RNA-seq datasets, like those from the UCSC Treehouse Childhood Cancer group (https://treehouse.cells.ucsc.edu).

## 3 Comparison to other available tools

Data from single-cell experiments can be visualized using a number of tools in addition to the visualization options available through the analysis packages Scanpy (Wolf *et al.*, 2018) and Seurat (Butler *et al.*, 2018; Stuart *et al.*, 2019). A recent paper (Çakır *et al.*, 2020) compares and contrasts 13 different solutions including the UCSC Cell Browser. Features that set ours apart from these are the ability to host many datasets on a single instance arranged as a hierarchy, a simple installation procedure that requires no special server infrastructure (e.g. Flask, Shiny), and built-in converters for many data formats.

## 4 Setting up an instance

A cell browser can be built from plain text files (expression matrix, metadata annotations, layout coordinates), but we provide utilities to import data from Seurat, Scanpy, Cellranger (Zheng *et al.*, 2017), Loom (http://loompy.org/) and other files. Both Seurat and Scanpy provide functions to export data and build a Cell Browser instance. Installation instructions and other documentation are available at https://cellbrowser.readthedocs.io/.

The UCSC Cell Browser can be used as a Galaxy, https://galaxyproject.org/ (Afgan *et al.*, 2018), tool for data analyzed or imported into an instance where it has been installed. The module can also be used at the public Human Cell Atlas (HCA) Galaxy instance at https://humancellatlas.usegalaxy.eu/, where it can be used to visualize reanalyzed data from the HCA (Regev *et al.*, 2017) and the Single Cell Expression Atlas (Papatheodorou *et al.*, 2020), as part of the SCiAp setup (Moreno *et al.*, 2021). The Bioconda (Grüning *et al.*, 2018) module has been installed more than 3.4k times and the Galaxy module, https://toolshed.g2.bx.psu.edu/view/ebi-gxa/ucsc_cell_browser, has been cloned to ∼185 Galaxy instances around the world.

## 5 Recent developments and future work

The website currently has 378 single-cell datasets arranged into 136 top-level projects, over 90 of which were added in the last 12 months (Supplementary Table S1). Our ATAC-seq support has been expanded in the past few months, with the ability to search genes and select nearby peaks for coloring (Supplementary Fig. S2). We have added the ability to associate large microscopy images with a dataset (Supplementary Fig. S3). During the next few months, we plan to further improve ATAC-seq support and custom annotations. Over the next year, we intend to add support for running analysis algorithms on selected cells on-the-fly.

## Funding

This work was supported by the National Human Genome Research Institute [5U41HG002371 to M.H., W.J.K., M.L.S., B.J.R., 1U41HG010972 to M.H., 5R01HG010329 to W.J.K.]; National Institutes of Health [U01MH114825 to W.J.K., K99 NS111731 to A.B., RF1MH121268 to T.J.N.]; National Institutes of Mental Health [DP2MH122400 to A.A.P.]; Silicon Valley Community Foundation [2017-171531(5022) to M.H., W.J.K., M.L.S.]; California Institute for Regenerative Medicine [GC1R-06673-C to M.H., W.J.K., M.L.S., GC1R-06673-B to L.S.]; University of California Office of the President Emergency COVID-19 Research Seed Funding [R00RG2456 to M.H.]; Chan Zuckerberg Initiative Foundation [CZF2019-002438 to N.S.M., 2018-183498 to P.M., I.P., 2018-182800 to L.S.]; Simons Foundation [SFARI 491371 to T.J.N.]; Brain and Behavior Research Foundation [NARSAD Young Investigator Grant to T.J.N]; Gifts from Schmidt Futures and the William K. Bowes Jr Foundation to T.J.N.

*Conflict of Interest*: none declared.

## Data availability

No new data were generated or analysed in support of this research.

## Supplementary Material

btab503_Supplementary_Data
