# Supplementary material for: UCSC Cell Browser: visualize your single-cell data
Source: Bioinformatics. 2021 Jul 9;37(23):4578–80. doi: 10.1093/bioinformatics/btab503 (PMC8652023; doi:10.1093/bioinformatics/btab503)
Supplement: btab503_Supplementary_Data [file btab503_supplementary_data.zip › UCSC_Cell_Browser_Fig_S1.pdf]

Supplemental Figures and Tables:

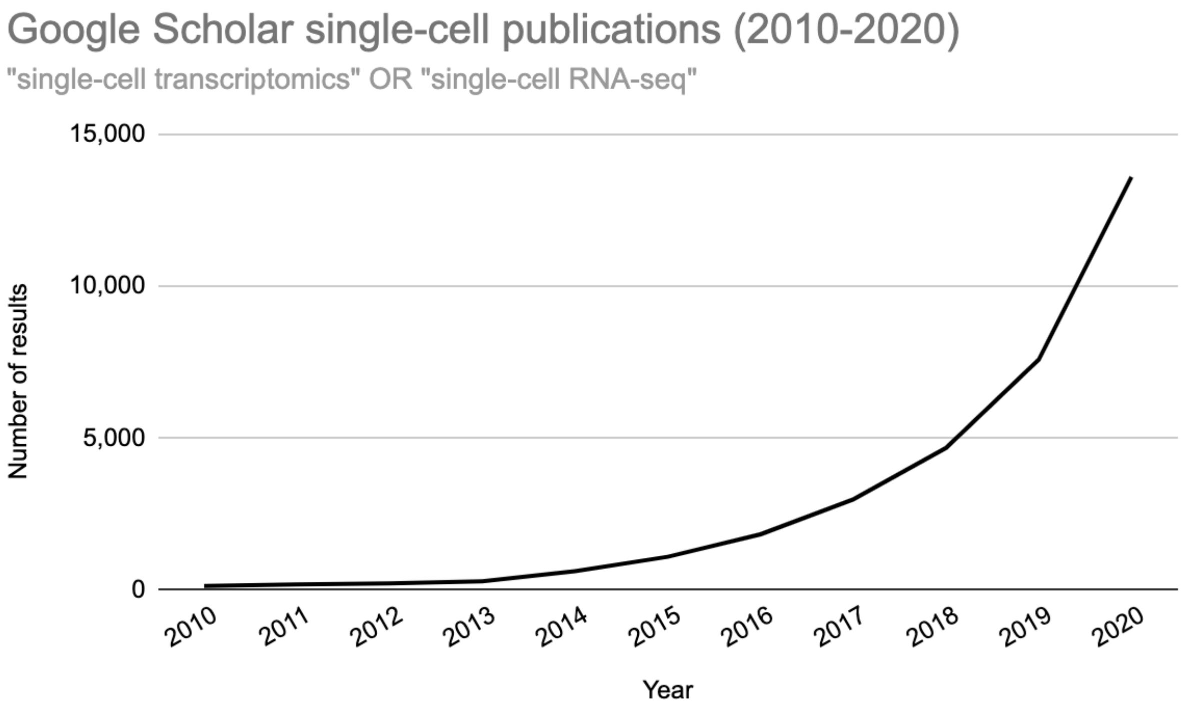

Figure S1: Single-cell publications in Google Scholar. The number of publications in Google Scholar matching a search for '"single-cell transcriptomics" OR "single-cell RNA-seq"' for the years 2010 - 2020.
