# Supplementary material for: UCSC Cell Browser: visualize your single-cell data
Source: Bioinformatics. 2021 Jul 9;37(23):4578–80. doi: 10.1093/bioinformatics/btab503 (PMC8652023; doi:10.1093/bioinformatics/btab503)
Supplement: btab503_Supplementary_Data [file btab503_supplementary_data.zip › UCSC_Cell_Browser_Fig_S2.pdf]

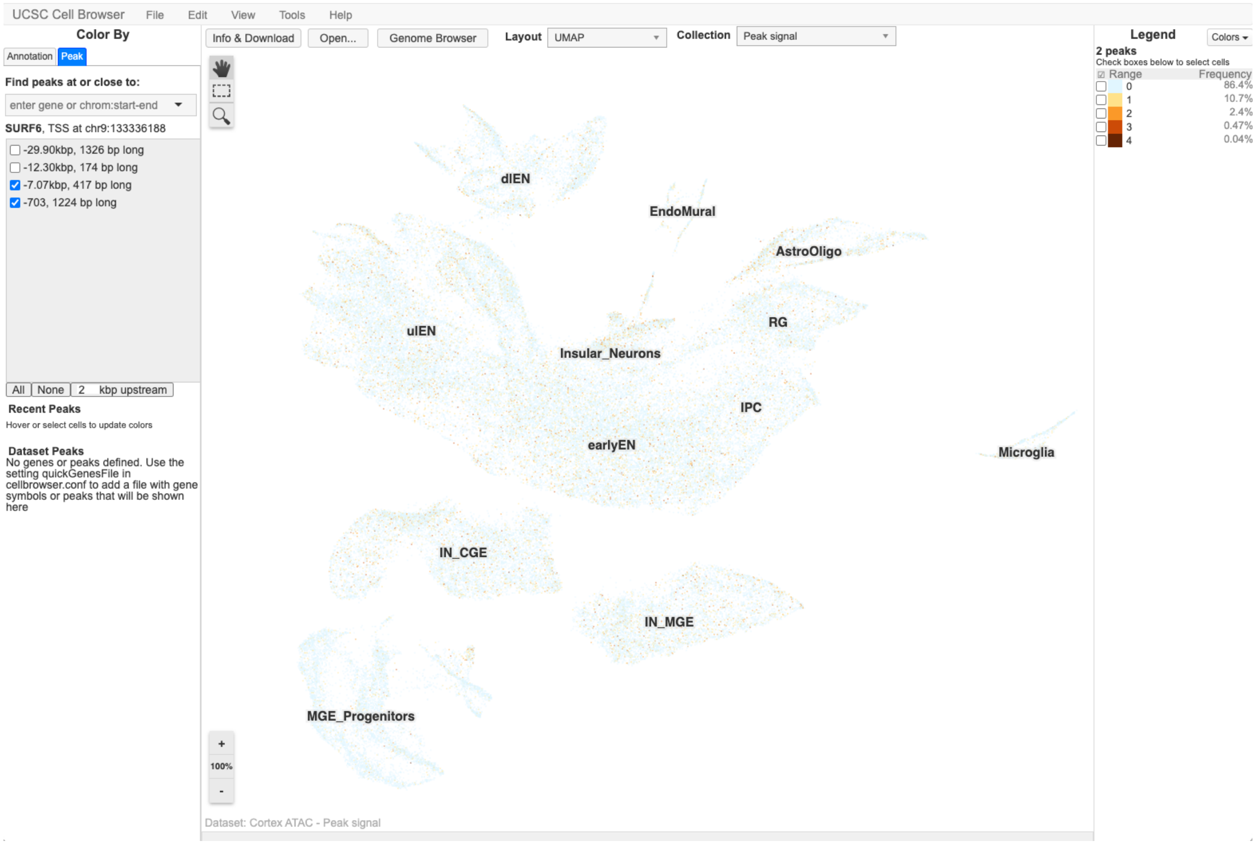

Figure S2: Single-cell ATAC-seq support in the UCSC Cell Browser. Search for genes or specific peaks in the box at the top left. Searching for a gene will result in a list of peaks being displayed with checkboxes to select those used for coloring the plot in the center of the screen. Peaks are sorted by their distance from the TSS of the currently selected gene.
