# Supplementary material for: UCSC Cell Browser: visualize your single-cell data
Source: Bioinformatics. 2021 Jul 9;37(23):4578–80. doi: 10.1093/bioinformatics/btab503 (PMC8652023; doi:10.1093/bioinformatics/btab503)
Supplement: btab503_Supplementary_Data [file btab503_supplementary_data.zip › UCSC_Cell_Browser_Fig_S3.pdf]

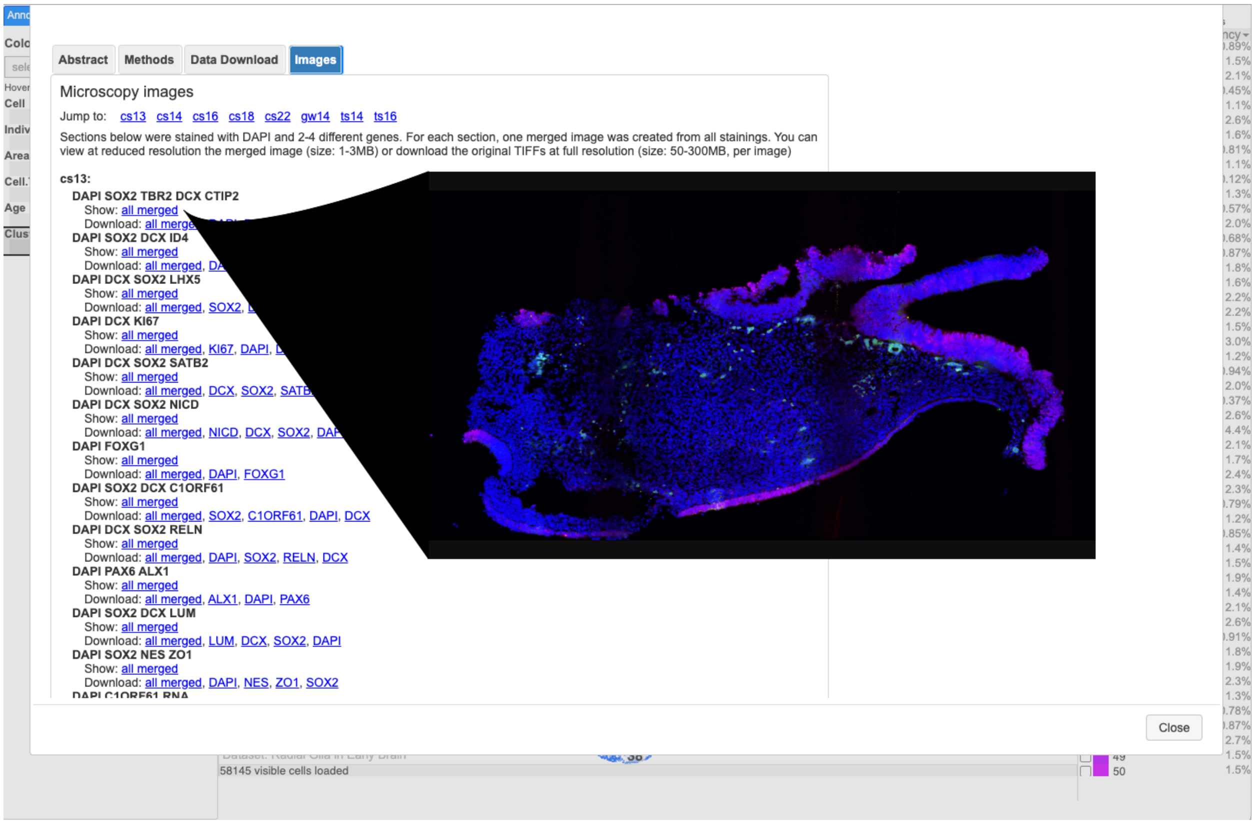

Figure S3: Microscopy image support in the UCSC Cell Browser. Microscopy images can also be associated with cell browser datasets. For example, in the “Radial Glia in Early Brain” dataset, https://early-brain.cells.ucsc.edu, there are fluorescently labeled images from each stage available. The “All Merged” link will bring up a JPEG (shown overlaid here) in a new tab, with high-quality TIFFs being available through the “Download” links below that.
