# Supplementary material for: UCSC Cell Browser: visualize your single-cell data
Source: Bioinformatics. 2021 Jul 9;37(23):4578–80. doi: 10.1093/bioinformatics/btab503 (PMC8652023; doi:10.1093/bioinformatics/btab503)
Supplement: btab503_Supplementary_Data [file btab503_supplementary_data.zip › UCSC_Cell_Browser_Table_S1.pdf]

Table S1: Datasets added in the last year. A table covering nearly 70 datasets that have been added to the UCSC Cell Browser in the last year publicly-available. The table doesn't include the 12 datasets that have been added that are still unpublished and not publicly-available at this time.

Dataset Name		Organism	Publication
New, publicly-available datasets added July 2020 - June 2021			
Colorectal Cancer	https://colorectal-cancer.cells.ucsc.edu	Human	Lee et al. 2020. Nat Genet.
Gut Cell Survey	https://gut-cell-atlas.cells.ucsc.edu	Human	Elmentaite et al. 2020. Dev Cell., James et al. 2020. Nat Immunol.
Human Foveal vs Peripheral Retina	https://human-fovea-periphery.cells.ucsc.edu	Human	Voigt et al. 2019. Exp Eye Res.
Human fetal pancreas and spheroids	https://human-pancreas-dev.cells.ucsc.edu	Human	Unpublished
ATAC-seq of Developing Human Cortex	https://cortex-atac.cells.ucsc.edu	Human	Unpublished
Tabula Sapiens	https://tabula-sapiens.cells.ucsc.edu	Human	Unpublished
Healthy Human Skin	https://healthy-human-skin.cells.ucsc.edu	Human	Reynolds et al. 2021. Science.
Fetal Chromatin Landscape	https://fetal-chromatin-atlas.cells.ucsc.edu	Human	Domcke et al. 2020. Science.
Fetal Gene Expression Atlas	https://fetal-gene-atlas.cells.ucsc.edu	Human	Cao et al. 2020. Science.
Risk Loci in Parkinson's and Alzheimer's	https://neuro-degen-atac.cells.ucsc.edu	Human	Corces et al. 2020. Nat Genet.
Aging Human Skin	https://aging-human-skin.cells.ucsc.edu	Human	Solé-Boldo et al. 2020. Commun Biol.
Endometrium During Menstrual Cycle	https://endometrium-cycle.cells.ucsc.edu	Human	Wang et al, Nat Med, 2020.
Cerebellar Development	https://cbl-dev.cells.ucsc.edu	Human	Aldinger et al. 2020. bioRxiv.
Cardiac Differentiation and TBX5	https://cardiac-differentiation.cells.ucsc.edu	Human	Kathiriya et al. 2020. Dev Cell.
X-chromosome Dosage in Preimplantation Embryos	https://preimplant-embryos.cells.ucsc.edu	Human	Petropoulos et al. 2016. Cell.
Developing Brain Lipid Heterogeneity	https://brain-lipids.cells.ucsc.edu	Human	Unpublished
Proximal Airway in Healthy and Cystic Fibrosis Patients	https://airway-cf.cells.ucsc.edu	Human	Carraro et al. 2020. bioRxiv.
Heart Cell Atlas	https://heart-cell-atlas.cells.ucsc.edu	Human	Litviňuková et al. 2020. Nature.
Integrated Analysis of Multimodal PBMC data	https://multimodal-pbmc.cells.ucsc.edu/	Human	Hao et al. 2020. bioRxiv.
Human Skeletal Muscle Atlas	https://muscle-cell-atlas.cells.ucsc.edu	Human	De Micheli et al. 2020. Skelet Muscle.
Lung Airway Epithelium	https://lung-airway.cells.ucsc.edu	Human	Okuda et al. 2020. Am J Respir Crit Care Med.

Leptomeningeal Metastasis	https://lepto-metastasis.cells.ucsc.edu	Human	Chi et al. 2020. Science.
Engraftable Hematopoietic Stem Cells	https://engraftable-hsc.cells.ucsc.edu	Human	Vanuytsel et al. 2020. bioRxiv.
Human Macrophage Development	https://macrophage-dev-bian2020.cells.ucsc.edu	Human	Bian et al. 2020. Nature.
Human Cellular Landscape via Microwell-Seq	https://human-cellular-landscape.cells.ucsc.edu	Human	Han et al. 2020. Nature.
Modeling Embryo Development using hPSCs	https://hpsc-embryo-model.cells.ucsc.edu	Human	Zheng et al. 2019. Nature.
Lung in Pulmonary Fibrosis and Control	https://lung-pf-control.cells.ucsc.edu	Human	Habermann et al. 2020. Sci Adv.
Radial Glia in Early Brain	https://early-brain.cells.ucsc.edu	Human	Eze et al. 2020. bioRxiv.
Vasculature in the Developing Brain	https://vascular-dev.cells.ucsc.edu	Human	Unpublished
Choroid Plexus Organoids	https://chporg.cells.ucsc.edu	Human	Pellegrini et al. 2020. Science.
Adult Testis	https://adult-testis.cells.ucsc.edu	Human	Guo et al. 2018. Cell Res.
Fetal Lung	https://fetal-lung.cells.ucsc.edu	Human	Miller et al. 2020. Dev Cell.
Human Ileum	https://human-ileum.cells.ucsc.edu	Human	Martin et al. 2019. Cell.
Human Colon in Ulcerative Colitis	https://human-colon.cells.ucsc.edu	Human	Smillie et al. 2019. Cell.
Human Intestine	https://human-intestine.cells.ucsc.edu	Human	Wang et al. 2020. J Exp Med.
Maternal-Fetal Interface (Placenta/Decidua)	https://placenta-decidua.cells.ucsc.edu	Human	Vento-Tormo et al. 2018. Nature.
Human Healthy Airways	https://healthy-human-airway.cells.ucsc.edu	Human	Deprez et al. 2019. bioRxiv.
Prostate and Prostatic Urethra	https://prostate-prostatic-urethra.cells.ucsc.edu	Human	Henry et al. 2018. Cell Rep.
DroNc-seq of Human Brain	https://brain-dronc-seq.cells.ucsc.edu	Human	Habib et al. 2017. Nat Methods.
Human Liver	https://human-liver.cells.ucsc.edu	Human	MacParland et al. 2018. Nat Commun.
Oral Cavity	https://oral-cavity.cells.ucsc.edu	Human	Huang et al. 2021. medRxiv.
Retinal Pigment Epithelium	https://retinal-pigment-epi.cells.ucsc.edu	Human	Voigt et al. 2019. PNAS.
Fetal Thymus	https://fetal-thymus.cells.ucsc.edu	Human	Park et al. 2020. Science.
Adult Retina	https://adult-retina.cells.ucsc.edu	Human	Lukowski et al. 2019. EMBO J.
Normal and Inflamed Human Epidermis	https://human-epidermis.cells.ucsc.edu	Human	Cheng et al. 2018. Cell Rep.
Human Pancreas	https://human-pancreas.cells.ucsc.edu	Human	Baron et al. 2016. Cell Syst.
Fetal Liver	https://fetal-liver.cells.ucsc.edu	Human	Popescu et al. 2019. Nature.
Human Cornea	https://human-cornea.cells.ucsc.edu	Human	Collin et al. 2021. The Ocular Surface.

Lung, Spleen, and Oesophagus Cryo-preservation Study	https://tissue-stability.cells.ucsc.edu		Madissoon et al. 2019. Genome Biol.
Gallbladder (Sampaziotis et al. Unpublished.)	https://human-gallbladder.cells.ucsc.edu	Human	Unpublished
Tumor Compendium V11 to the Treehouse Collection	https://treehouse.cells.ucsc.edu	Human	Multiple
Human and Mouse Dental Cells	https://dental-cells.cells.ucsc.edu	Human + Mouse	Krivanek et al. 2020. Nat Commun.
Allen Brain Map: Celltypes Database	https://allen-celltypes.cells.ucsc.edu	Human + Mouse	Multiple
Mouse DRG Injury	https://mouse-drg-injury.cells.ucsc.edu	Mouse	Avraham et al. 2020. Nat Commun.
Mouse Mammary Epithelium	https://mouse-mammary-epithelium-integrated.cells.ucsc.edu	Mouse	Unpublished
Histone Profiling in Mouse Brain	https://mouse-brain-cutandtag.cells.ucsc.edu	Mouse	Bartosovic et al. 2020. bioRxiv.
Oligodendrocyte Heterogeneity in Mouse	https://mouse-oligo-het.cells.ucsc.edu	Mouse	Marques et al. 2016. Science.
Oligodendrocyte Lineage in Developing Mouse Brain	https://oligo-lineage-dev.cells.ucsc.edu	Mouse	Marques et al. 2018. Dev Cell.
Oligodendrocyte Lineage in Multiple Sclerosis	https://oligo-lineage-ms.cells.ucsc.edu	Mouse	Mendanha Falcão et al. 2018. Nat Med.
Mouse Embryonic Forelimb	https://mouse-limb.cells.ucsc.edu	Mouse	He et al. 2020. Nature.
Mouse Hypothalamus Development	https://mouse-hypothalamus-dev.cells.ucsc.edu	Mouse	Romanov et al. 2020. Nature.
Mouse Embryonic Pancreas	https://mouse-embryonic-pancreas.cells.ucsc.edu	Mouse	Gurp et al. 2019. Development.
Skin Stretch Response in Mouse	https://mouse-skin-stretch.cells.ucsc.edu	Mouse	Aragona et al. 2020. Nature.
SVZ Neurogenic Lineage in Mouse	https://svzneurogeniclineage.cells.ucsc.edu	Mouse	Redmond et al. 2020. bioRxiv.
Molecular Architecture of the Mouse Nervous System	https://mouse-nervous-system.cells.ucsc.edu	Mouse	Zeisel et al. 2018. Cell.
Mouse Esophagus	https://mouse-esophagus.cells.ucsc.edu	Mouse	Kabir et al. 2021. bioRxiv.
Developing Mouse Brain	https://mouse-dev-brain.cells.ucsc.edu	Mouse	Manno et al. 2020. bioRxiv.
Mouse Cell Atlas via Microwell-Seq	https://mouse-cell-atlas.cells.ucsc.edu	Mouse	Han et al. 2018. Cell.
Developing Mouse Neocortex	https://mouse-dev-neocortex.cells.ucsc.edu	Mouse	Li et al. 2020. Sci Adv.
Regulation in Mouse	https://mouse-kidney-atac.cells.ucsc.edu	Mouse	Miao et al. 2020. bioRxiv.

Kidney			
Sepsis Timeline in Mouse Kidney	https://mouse-kidney-sepsis.cells.ucsc.edu	Mouse	Janosevic et al. 2021. eLife.
Atlas of Zebrafish Neural Crest	https://zebrafish-neural-crest-atlas.cells.ucsc.edu	Zebrafish	Howard et al. 2020. bioRxiv.
Neurons of the Drosophila Visual System	https://dros-visual-sys.cells.ucsc.edu	Fruit Fly	Özel et al. 2020. Nature.
Drosophila Olfactory Neurons	https://dros-olfac.cells.ucsc.edu	Fruit Fly	McLaughlin et al. 2020. eLife.
Drosophila Ovary	https://dros-ovary.cells.ucsc.edu	Fruit Fly	Jevitt et al. 2020. PLoS Biol.
Sea Urchin Development	https://sea-urchin-dev.cells.ucsc.edu	S. purpuratus	Foster et al. 2020. Development.
Sea Urchin Pigment Cells	https://sea-urchin-pigment.cells.ucsc.edu	S. purpuratus	Perillo et al. 2020. eLife.
Single-cell Atlas of Parasite S. mansoni	https://s-masoni.cells.ucsc.edu	S. mansoni	Wendt et al. 2020. Science.
Five datasets from the Alexandria Project	https://shalek-alexandria-project.cells.ucsc.edu	Multiple	Multiple
COVID-19 Datasets added July 2020 - June 2021			
Airway Epithelium-Immune Interactions in COVID-19	https://covid-airways.cells.ucsc.edu	Human	Chua et al. 2020. Nat Biot.
A Map of Human Coronavirus Entry Factors	https://scarface.cells.ucsc.edu	Human	Singh et al. 2020. Cell Rep.
Cellular Targets of SARS-CoV-2	https://covid19-cellular-targets.cells.ucsc.edu	Human	Delorey et al. 2021. bioRxiv.
SARS-CoV-2 and Cigarette Smoke	https://covid19-smoking.cells.ucsc.edu	Human	Purkayastha et al. 2020. Cell Stem Cell.
Bronchoalveolar Immune Cells in COVID-19	https://covid19-balf.cells.ucsc.edu	Human	Liao et al. 2020. Nat Med.
COVID-19 and Influenza Immunophenotyping	https://covid19-influenza-response.cells.ucsc.edu	Human	Lee et al. 2020. Sci Immunol.
Cytokine Storm in COVID-19	https://covid19-cytokine-storm.cells.ucsc.edu	Human	Guo et al. 2020. Nat Commun.
COVID-19 in Hypertensive Patients	https://covid-hypertension.cells.ucsc.edu	Human	Trump et al. 2020. medRxiv.
Immunological Response in COVID-19 Patients	https://covid19-immuno.cells.ucsc.edu	Human	Zhang et al. 2020. Nat Immunol.
Immunological Landscape in Critical COVID-19	https://covid19-critical-immuno.cells.ucsc.edu	Human	Ren et al. 2020. bioRxiv.
SARS-Cov-2 Target Cells in Human Airway	https://covid19-bronch-epi.cells.ucsc.edu	Human	Ravindra et al. 2021. PLoS Biol.
Immune Response in Fatal COVID-19 Patients	https://covid19-fatal.cells.ucsc.edu	Human	Liu et al. 2021. Cell.
